# Global Initiative for Childhood Cancer Control: Increasing access, improving quality, saving lives

**DOI:** 10.1590/1518-8345.0000.3999

**Published:** 2023-10-06

**Authors:** Regina Aparecida Garcia de Lima, Luís Carlos Lopes-Júnior, Edmara Bazoni Soares Maia, Soad Fuentes-Alabi, María Liliana Vásquez Ponce

**Affiliations:** 1 Universidade de São Paulo, Escola de Enfermagem de Ribeirão Preto, Centro Colaborador da OPAS/OMS para o Desenvolvimento da Pesquisa em Enfermagem, Ribeirão Preto, SP, Brasil.; 2 Universidade Federal do Espírito Santo, Centro de Ciências da Saúde, Vitória, ES, Brasil.; 3 Universidade Federal de São Paulo, Escola Paulista de Enfermagem, Departamento de Enfermagem Pediátrica, São Paulo, SP, Brasil.; 4 Organización Panamericana de la Salud, Departamento de Enfermedades No Transmisibles, Unidad de Cancer, Washington, D.C., Estados Unidos de América.; 5 Pan American Health Organization, Unit of Non Communicable Diseases, Washington, D.C., Estados Unidos de América.

**Figure:**
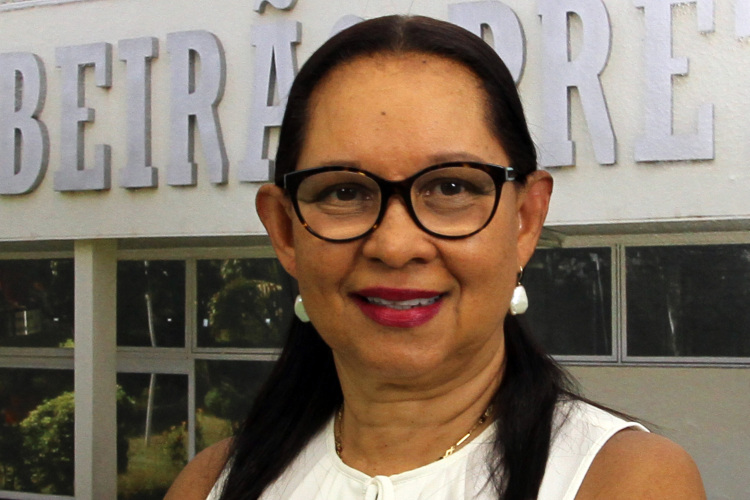

**Figure:**
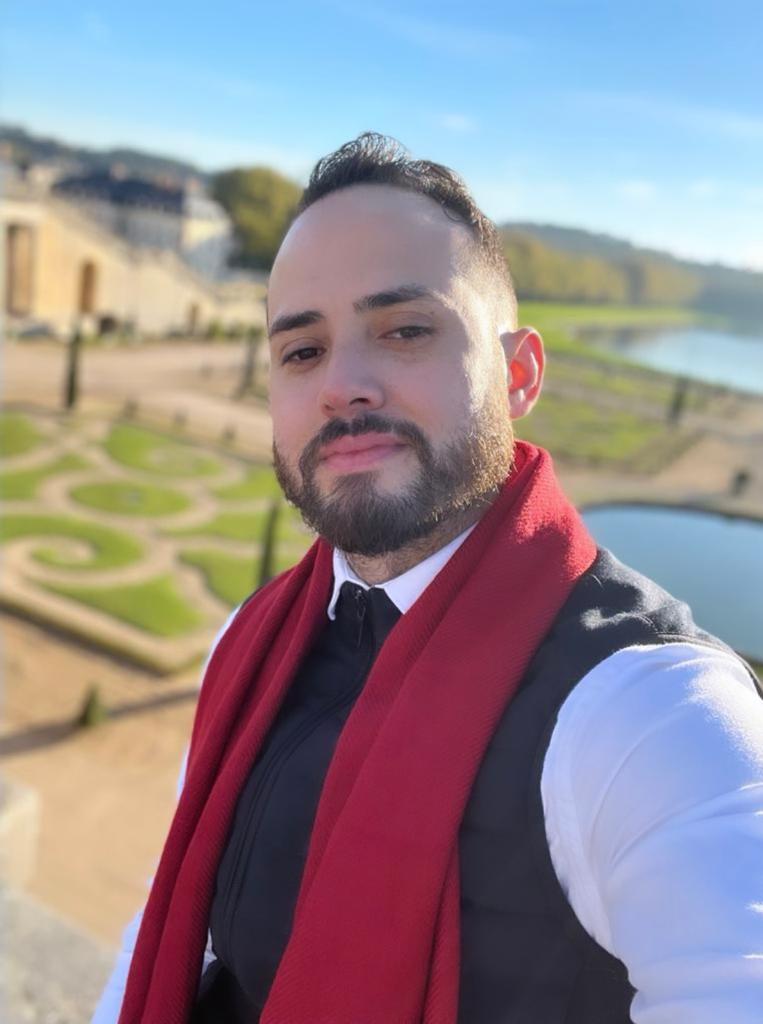

**Figure:**
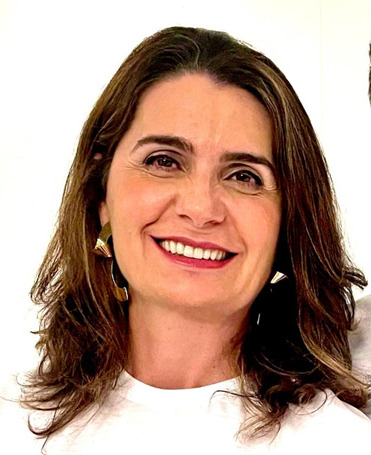

**Figure:**
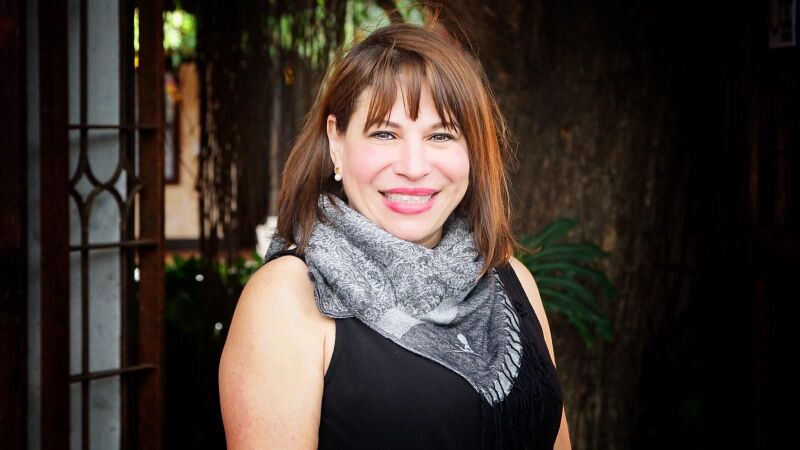

**Figure:**
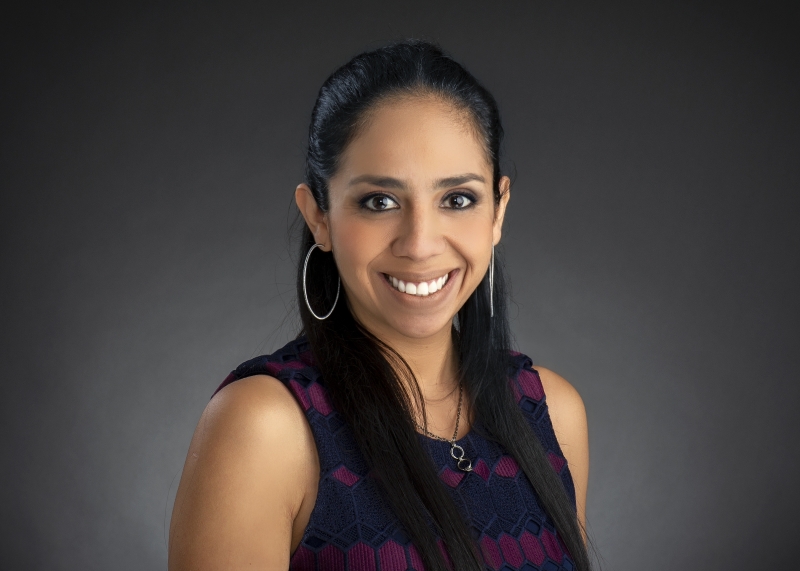

The global cancer statistics are increasingly alarming: in 2022 there were approximately 20 million new cancer cases and 10 million deaths^(^ 1 ^)^. In particular, regarding the childhood cancer, nearly 280,000 children and adolescents aged from 0 to 19 years old develop the disease each year in the world^(^ 2 ^)^ and, of them, 9 out of 10 live in low- and middle-income countries, where treatments are generally not available or present high costs^(^ 2 ^)^. In Latin America and the Caribbean, it is estimated that at least 29,000 individuals aged less than 19 years old will develop cancer each year. In high-income countries, more than 80% of the children and adolescents with cancer are cured; however, this rate is approximately 20% in low- and middle-income countries^(^ 3 ^)^.

The preventable deaths due to cancer in this population group from low- and middle-income countries are related to underdiagnosis, late or incorrect diagnosis, health care access difficulties, advanced-stage disease at the time of diagnosis, and lack of access to treatments and supportive care in reference centers or to health professionals with specialized knowledge and training. They can also be due to treatment abandonment as a result of the families’ difficulties bearing the direct health care costs, to deaths due to drug toxicities and to higher recurrence rates. Underlying reasons for this disparity have been linked to health system indicators such as annual *per capita* national expenditure in health, number of physicians and nurses *per* 1,000 inhabitants and institutional capacity. Regarding the institutional capacity, inadequate staffing of duly qualified health professionals and limited supportive care resources stand out, such as medications and hemoderivatives^(^ 3 ^)^.

Even if considered rarer than cancer in adults, it also emerges as a relevant public health issue^(^ 2 ^)^. Investing in cancer control programs, in particular childhood cancer, overcomes the economic costs, as each child or adolescent who dies prematurely due to the disease represents a loss for the family and a threat to family cohesion, also leading to a long-term social loss. In addition to the economic reason, the policies for childhood cancer control might be incorporate the equality, human rights and social justice principles^(^ 3 ^-^ 4 ^)^. Such principles are supported by the Alma-Ata Declaration^(^ 5 ^)^ when it deals with valuing human life and dignity, asserting that all people are important and cannot be left behind. The crucial need for equality in cancer care, regardless of age, sociodemographic background or diagnosis, is the basis for social justice in healthcare services^(^ 4 ^)^. Hence, a child’s or adolescent’s place of residence should be not determine if they will survive cancer or not.

In order to face this profound inequality, in 2018 the World Health Organization (WHO) and the St. Jude Children’s Research Hospital launched the WHO Global Initiative for Childhood Cancer Control and, to achieve the objective proposed of increasing survival of children and adolescents with cancer to at least 60% by 2030, a number of improvements in care access and quality were established^(^ 3 ^)^. Thus, the Initiative have defined two main objectives: increasing the countries capability to provide good quality services and information; and increasing the priority of childhood cancer at the global, national and regional levels, based on the CureAll framework^(^ 3 ^)^.

The CureAll structure highlights the need for multidisciplinary care centered on the families of children and adolescents with cancer. It is expected that the development of resources targeted at healthcare professionals as a pivotal element to ensure the implementation of evidence-based practices in cancer care. Such resources, including guidelines, training courses and technical reports, provide healthcare professionals with the tools and information required to delivering good quality care adjusted to their patients’ needs.

Several strategies have been proposed to improving the survival rate of children and adolescents with cancer in low- and middle-income countries, including efforts to raise awareness in the communities, care guided by cooperative protocols, and interdisciplinary care. The need for competent Pediatric Oncology Nurses is a care component universally recognized as essential in all these strategies^(^ 6 ^)^.

The WHO recognizes nurses as “front-line clinicians”, essential to improve healthcare access^(^ 7 ^)^. In this sense, specialized education, adequate staffing and devices in sufficient numbers are necessary to support the complex nursing practice. Pediatric oncology nursing care requires broad and deep knowledge about childhood cancer and advanced clinical skills. In high-income countries, hospitals provide permanent education to recently-hired pediatric oncology nurses, unlike the reality in health institutions from low and middle-income countries, where this is oftentimes not available. In addition, in many countries, nurses’ professional career plans are not always structured to regulate Oncology nurses’ role and functions and, ultimately, financial support according to their competencies^(^ 6 ^-^ 7 ^)^.

Hence, the recent technical report entitled “Scope of Pediatric Oncology Nursing Practice in Latin America”^(^ 8 ^)^, as part of the CureAll framework, launched in January 2023 by the Pan American Health Organization (PAHO), carries with it the unprecedented fact of gathering and systematizing the recommendations to support nurses from Latin America and the Caribbean in their duty of caring for children and adolescents with cancer and their families. This technical report is intended for healthcare and hospital managers, for training institutions and for Oncology Nursing, mainly nurses specialized in Pediatric Oncology, with the purpose of presenting the Essential Competencies for the Practice of Pediatric Oncology Nurses in Latin America and the Caribbean. The theoretical grounds to systematize the recommendations comprised in this technical report Patient- and Family-Centered Care, an internationally renowned and widely used framework with a theoretical structure that enables to bond between different realities of this practice. The second theoretical basis was “Caring for Teenagers and Young Adults with Cancer: a Competence and Career Framework for Nursing”, prepared by the Teenage Cancer Trust/Royal College of Nursing. This document presents the process to build nurses’ competencies for the care of adolescents and young adults with cancer, supported by six domains: 1) Clinical and supportive care; 2) Education and research; 3) Involvement and advocacy; 4) Interprofessional team and the itinerary of children and adolescents with cancer and their families; 5) Leadership and professional development; and 6) Development of health policies and services.

Both theoretical bases are also in line with the WHO Global Initiative for Childhood Cancer Control guidelines in terms of the need for excellence centers and care networks with specialized interprofessional teams and collaborative practices. Thus, permanent education and training are key elements to enhance Pediatric Oncology nurses’ skills, clinical knowledge, leadership and political capability.

Healthcare professionals, especially nurses, educators and stakeholders need to identify and incorporate the essential competencies that determine the scope of a safe and qualified cancer care practice based on knowledge, skills and attitudes, necessary characteristics for an effective professional clinical practice. Identifying these competencies ensures that healthcare professions are well-defined, promotes competent workforces, eases timely, autonomous and scientific assessments through evidence-based practices and eases professional mobility.

The WHO Global Initiative for Childhood Cancer Control is a public policy that presents Pediatric Oncology Nurses with guidelines so that they can change the circumstances where they want to work, in order to achieve the goal of improving the survival rate of children and adolescents with cancer in the next decades.
